# Dialysis Disequilibrium Syndrome: Brain death following hemodialysis for metabolic acidosis and acute renal failure – A case report

**DOI:** 10.1186/1471-2369-5-9

**Published:** 2004-08-19

**Authors:** Sean M Bagshaw, Adam D Peets, Morad Hameed, Paul JE Boiteau, Kevin B Laupland, Christopher J Doig

**Affiliations:** 1 Department of Critical Care Medicine, Calgary Health Region and University of Calgary, Calgary, Alberta, Canada; 2 Department of Community Health Sciences, Calgary Health Region and University of Calgary, Calgary, Alberta, Canada; 3 Department of Surgery, Calgary Health Region and University of Calgary, Calgary, Alberta, Canada; 4 Department of Medicine, Calgary Health Region and University of Calgary, Calgary, Alberta, Canada; 5 Department of Diagnostic and Laboratory Medicine, Calgary Health Region and University of Calgary, Calgary, Alberta, Canada

## Abstract

**Background:**

Dialysis disequilibrium syndrome (DDS) is the clinical phenomenon of acute neurologic symptoms attributed to cerebral edema that occurs during or following intermittent hemodialysis (HD). We describe a case of DDS-induced cerebral edema that resulted in irreversible brain injury and death following acute HD and review the relevant literature of the association of DDS and HD.

**Case Presentation:**

A 22-year-old male with obstructive uropathy presented to hospital with severe sepsis syndrome secondary to pneumonia. Laboratory investigations included a pH of 6.95, PaCO2 10 mmHg, HCO3 2 mmol/L, serum sodium 132 mmol/L, serum osmolality 330 mosmol/kg, and urea 130 mg/dL (46.7 mmol/L). Diagnostic imaging demonstrated multifocal pneumonia, bilateral hydronephrosis and bladder wall thickening. During HD the patient became progressively obtunded. Repeat laboratory investigations showed pH 7.36, HCO3 19 mmol/L, potassium 1.8 mmol/L, and urea 38.4 mg/dL (13.7 mmol/L) (urea-reduction-ratio 71%). Following HD, spontaneous movements were absent with no pupillary or brainstem reflexes. Head CT-scan showed diffuse cerebral edema with effacement of basal cisterns and generalized loss of gray-white differentiation. Brain death was declared.

**Conclusions:**

Death is a rare consequence of DDS in adults following HD. Several features may have predisposed this patient to DDS including: central nervous system adaptations from chronic kidney disease with efficient serum urea removal and correction of serum hyperosmolality; severe cerebral intracellular acidosis; relative hypercapnea; and post-HD hemodynamic instability with compounded cerebral ischemia.

## Background

Acute renal failure requiring hemodialysis (HD) is a common clinical problem in critically ill patients that is independently associated with increased mortality[1]. Dialysis disequilibrium syndrome (DDS) is the clinical phenomenon of acute central nervous system dysfunction attributed to cerebral edema that occurs during or following HD. The precise epidemiology of DDS is poorly defined[2]. Review of MEDLINE (January 1966 – March 2004) suggested that DDS in critically ill patients has rarely been reported[3,4]. We report a case of DDS-induced cerebral edema that resulted in irreversible brain injury and death following acute HD. Further, we review the relevant literature of the association of DDS and HD.

## Case presentation

A 22-year-old homosexual male presented to hospital with progressive dyspnea, productive cough, generalized malaise and fever. He had a known history of intravenous cocaine abuse and recent serology in prior 3 months was negative for human immunodeficiency virus (HIV). Results of a physical examination showed signs of tachypnea, tachycardia, accessory muscle use and left lung base crackles. Tympanic temperature was 34.7°C. The remainder of the examination was unremarkable except for urethral meatus stenosis.

Initial laboratory investigations are presented in Table 1. Arterial blood gases showed pH of 6.95, PaCO_2 _10 mmHg, PaO_2 _109 mmHg, HCO_3 _2 mmol/L, and lactate 0.6 mmol/L consistent with high anion gap metabolic acidosis with respiratory compensation. Serum creatinine and blood urea nitrogen were 587 μmol/L and 46.7 mmol/L, respectively. Toxicology and drug screen was negative. The metabolic acidosis was partially accounted for by acute renal failure with retained unmeasured anions and ketonemia. Urinalysis showed pyuria. Electrocardiogram (ECG) showed normal sinus rhythm.

**Table 1:** Laboratory values at prior to and following initiation of hemodialysis in the intensive care unit.

**Laboratory test**	**Pre-dialysis Value**	**Post-dialysis Values**	**Reference range**
**Hemoglobin**	96	78	137–180 g/L
**White cell count**	25.8	16.1	4.0–11.0 × 10^9^/L
**Band count**	3.1	-	0.0–1.3 × 10^9^/L
**Platelets**	603	486	150–400 × 10^9^/L
**Sodium**	132	132	133–145 mmol/L
**Potassium**	3.1	1.8	3.5–5.0 mmol/L
**Chloride**	107	93	98–111 mmol/L
**Bicarbonate**	2	19	21–31 mmol/L
**Glucose**	6.3	9.0	3.6–11.1 mmol/L
**Magnesium**	0.88	0.57	0.65–1.15 mmol/L
**Osmolality**	330	-	280–300 mosmol/kg
**Urea**	46.7	13.7	3.0–7.6 mmol/L
**Creatinine**	537	-	61–111 μmol/L
**Lactate**	0.6	1.2	< 2.0 mmol/L
**Serum ketones**	2+	-	Undetected
**Anion gap**	23	20	12–14
**Osmolar gap**	14.5	-	0–10

Chest radiograph revealed right middle lobe and lingular patchy opacification. An abdomino-pelvic CT scan demonstrated moderate to severe bilateral hydronephrosis, bladder wall thickening with multiple diverticuli, and retroperitoneal streaking consistent with acute infection. A provisional diagnosis of severe sepsis was made with multiple potential foci of infection. The patient was given empiric ceftriaxone, metronidazole and vancomycin. Sputum specimen cultured heavy methicillin-sensitive *Staphylococcus aureus*, blood cultures were positive for *S. aureus*, *Escherichia coli*, and Group B *Streptococcus*. Urine cultured greater than 10^8 ^CFU/L of multiple gram positive and negative organisms.

**Figure 1:**
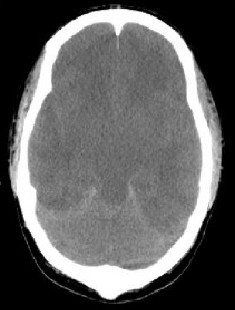
Computerized Tomography (CT) head showing diffuse cerebral edema with effacement of basal cisterns and generalized loss of gray-white differentiation

The patient was admitted to the intensive care unit (ICU). The metabolic acidosis persisted (pH 7.00) a despite 100 mEq of 8.4% sodium bicarbonate bolus and infusion of three liters of normal bicarbonate solution (150 mEq of 8.4% sodium bicarbonate in 1000 mL D5W). The patient had a suprapubic bladder catheter inserted by angiography. However, due to concern the patient remained oliguric following 4 L crystalloid resuscitation, hemodialysis was organized. Hemodialysis parameters included: F160 membrane (surface area 1.5 m^2 ^and KUf 50 mL/hr/mmHg), dialysate sodium 136 mmol/L, potassium 3 mmol/L, calcium 1.25 mmol/L, bicarbonate 40 mmol/L, and Q_D _500 mL/min, Q_B _250–300 mL/min via a 25 cm left femoral double-lumen Uldall catheter. The patient had 71.5 L of blood processed over four hours with no fluid removal. Although the patient was alert and appropriate (Glasgow Coma Scale 15) with tachycardia and stable normal range blood pressure before the initiation of dialysis, he was demonstrating an increased work of breathing and oxygen requirements suggestive of worsening sepsis syndrome. Approximately 2.5 hrs after start of dialysis the patient became rapidly unresponsive prompting intubation for airway protection. At completion of HD and over the subsequent 4 hours the patient's neurologic status deteriorated with evidence of loss of all brainstem reflexes. Head CT-scan is shown in Figure 1.

Repeat laboratory investigations immediately following hemodialysis revealed a pH 7.36, HCO3 19 mmol/L, sodium 132 mmol/L, potassium 1.8 mmol/L, and urea 13.7 mmol/L (urea-reduction-ratio was 71%) (Table 1).

The patient rapidly progressed to refractory shock and multi-organ dysfunction Diagnosis of brain death was declared independently by an intensivist and a neurologist. At autopsy, the brain showed evidence of diffuse cerebral edema. Cardiac assessment showed left ventricular enlargement consistent with systemic hypertension likely as a result of chronic kidney disease. Both lungs showed patchy acute bronchopneumonia with edema and congestion. Both kidneys appeared grossly pyonephrotic with dilated, thickened ureters and suggested the presence of acute on chronic pyelonephritis. The meatal aperture was scarred and stenosed.

## Discussion

The immediate indication for renal replacement therapy was correction of refractory metabolic acidosis in the setting of oliguria; however, following initiation of HD this patient developed irreversible symptoms consistent with DDS.

DDS occurs most commonly following initiation of chronic HD for patients with end-stage renal disease[2]. Patients with pre-existing neurologic disease, such as head trauma, stroke or malignant hypertension, may be at greater risk for developing DDS[5,6]. The precise epidemiology of DDS is poorly defined and may be under-reported due to the wide spectrum of clinical manifestations. Mild symptoms such as headache, nausea, blurred vision, muscle cramps, disorientation, anorexia, restlessness, hypertension and dizziness are common during or following HD and may be attributed to DDS[2]. More severe symptoms consistent with central nervous system dysfunction such as seizures, central pontine myelinolysis, coma and death are rare[7]. The temporal profile for DDS is not well described. DDS has been credited for acute electroencephalographic (EEG) abnormalities and structural changes on diagnostic imaging following rapid hemodialysis [8-10]. Likewise, brain MRI studies immediately following hemodialysis in chronic dialysis patients have shown quantitative increases in brain volume consistent with cerebral edema[11].

The pathogenesis remains debated and incompletely understood; however, two central hypotheses have emerged. First, acute urea removal occurs more slowly across the blood-brain barrier than from plasma, generating a 'reverse osmotic gradient' promoting water movement into the brain and cerebral edema[12]. Absolute increases in brain water content have been demonstrated in a rat model of uremia undergoing rapid hemodialysis that was accounted for by an increase in the ratio of brain to plasma urea[13,14]. Down-regulation of central nervous system urea transporters have been proposed as a mechanism contributing to the delay in urea clearance from the brain[15].

The second hypothesis states that the increased osmolality of the extracellular fluid in uremia stimulates an adaptive accumulation of intracellular organic osmolytes to limit cerebral cell dehydration[16]. During hemodialysis, retention of these organic osmolytes contributes to a paradoxical reduction in intracellular pH resulting in increased brain osmolality and cerebral edema[17,18].

The patient in this case unfortunately may have been susceptible to both proposed pathophysiologic mechanisms. The likely and under-appreciated presence of pre-existing kidney disease (chronic obstructive nephropathy and pyelonephritis) with an increased serum osmolality would have resulted in adaptive changes in the central nervous system. Ensuing hemodialysis correction of the plasma metabolic acidosis may have eclipsed a more severe cerebral intracellular acidosis. Further, urea clearance by hemodialysis was efficient at approximately 70% and probably generated a sufficient plasma-to-brain urea gradient for development of cerebral edema, intracranial hypertension and DDS. A less efficient initial course of hemodialysis would have diminished the osmolar gradient of urea across the central nervous system reducing the likelihood of symptoms of DDS.

Other variables may have contributed. The patient was compensating for the severe metabolic acidosis by hyperventilation (PaCO_2 _10 mmHg); however, initial post-intubation PaCO_2 _was 42 mmHg. Rapid elevations in PaCO_2 _can alter cerebral autoregulation resulting in exacerbated intracranial hypertension[19]. Concomitant sepsis syndrome with poly-microbial bacteremia resulting in widespread immune activation may alter blood-brain-barrier permeability and contribute to cerebral edema[20,21]. These factors likely contributed to an increased risk for DDS-induced cerebral edema.

The symptoms of DDS have been ameliorated by several interventions targeted to reduce the hemodialysis-induced plasma-to-brain osmotic gradient promoting cerebral edema[22]. A similar case of severe DDS requiring intubation was prevented from recurring during subsequent hemodialysis by use of modified dialysate containing 10.1 mmol/L of urea[23]. Likewise, the administration of intravenous mannitol and hyperventilation reversed a case of severe DDS-induced central nervous system dysfunction in a patient undergoing initial hemodialysis for acute renal failure[3]. Conversely, sodium profiling, high sodium or hyperglycemic dialysate have been attempted with variable results[24,25].

Prevention of DDS is traditionally the mainstay of therapy, particularly during initiation of hemodialysis in new patients. Despite the absence of evidence-based guidelines, the conventional aim is for a gradual clearance of urea. This can be accomplished with intermittent hemodialysis by use of a smaller, less efficient dialyzer and by reducing the duration of initial dialysis to approximately 2 hours with targeted lower blood flow rates of 150–200 mL/min, use of sustained low-efficiency dialysis (SLED), or initiation of continuous renal replacement therapy (CRRT) with more gradual and stable clearance of urea[2,26-28]. As a result, DDS has not been reported with the use of SLED or CRRT in critically ill patients. By providing a shorter, less efficient trial of initial hemodialysis, the severe DDS and brain death in this case may have been altogether prevented.

In summary, the precise epidemiology and pathophysiology of DDS remain unclear. Although DDS usually presents in end-stage renal disease patients undergoing initial therapy, critically ill patients may represent a unique population where co-existing illnesses such as sepsis, brain injury or other central nervous system disease, multiorgan dysfunction, and need for sedation can present obstacles for prompt diagnosis of DDS. Furthermore, for similar reasons, critically ill patients may have increased susceptibility to DDS conditions.

## Competing interests

None declared.

## Authors' contributions

SMB wrote and revised the manuscript. ADP, MH, PJEB, KBL and CJD provided critique of successive drafts of the manuscript. All authors read and approved the final manuscript.

## Pre-publication history

The pre-publication history for this paper can be accessed here:


